# Ten-year mixed-method evaluation of prelicensure health professional student self-reported learning in an interfaculty pain curriculum

**DOI:** 10.1097/PR9.0000000000001030

**Published:** 2022-09-14

**Authors:** Craig M. Dale, Iacopo Cioffi, Laura Murphy, Sylvia Langlois, Renata Musa, Bonnie Stevens

**Affiliations:** a Lawrence S. Bloomberg Faculty of Nursing, University of Toronto, Toronto, ON, Canada; b University of Toronto Centre for the Study of Pain, University of Toronto, Toronto, ON, Canada; c Tory Trauma Program, Sunnybrook Health Sciences Centre, Toronto, ON, Canada; d Faculty of Dentistry, Centre for Sensorimotor Multimodal and Pain Research, University of Toronto, Toronto, ON, Canada; e Leslie Dan Faculty of Pharmacy, University of Toronto, Toronto, ON, Canada; f Department of Pharmacy and KITE Research Institute, University Health Network, Toronto, ON, Canada; g Department of Occupational Science and Occupational Therapy, University of Toronto, Toronto, ON, Canada; h Centre for Interprofessional Education, University of Toronto, Toronto, ON, Canada; i Research Institute, The Hospital for Sick Children (SickKids), Toronto, ON, Canada

**Keywords:** Curriculum, Interprofessional education, IPE, Mixed methods, Pain, Prelicensure

## Abstract

**Introduction:**

Student perspectives on interprofessional pain education are lacking.

**Objectives:**

The purpose of this study was to evaluate ratings of knowledge acquisition and effective presentation methods for prelicensure health professional students attending the University of Toronto Centre for the Study of Pain Interfaculty Pain Curriculum (Canada).

**Methods:**

A 10-year (2009–2019) retrospective longitudinal mixed-methods approach comprising analysis and integration of quantitative and qualitative data sets was used to evaluate 5 core University of Toronto Centre for the Study of Pain Interfaculty Pain Curriculum learning sessions.

**Results:**

A total of 10, 693 students were enrolled (2009–2019) with a mean annual attendance of 972 students (±SD:102). The mean proportion of students rating “agree/strongly agree” for knowledge acquisition and effective presentation methods across sessions was 79.3% (±SD:3.4) and 76.7% (±SD:6.0), respectively. Knowledge acquisition or presentation effectiveness scores increased, respectively, over time for 4 core sessions: online self-study pain mechanisms module (*P* = 0.03/*P* < 0.001), online self-study opioids module (*P* = 0.04/*P* = 0.019), individually selected in-person topical pain sessions (*P* = 0.03/*P* < 0.001), and in-person patient or interprofessional panel session (*P* = 0.03). Qualitative data corroborated rating scores and expanded insight into student expectations for knowledge acquisition to inform real-world clinical practice and interprofessional collaboration; presentation effectiveness corresponded with smaller session size, individually selected sessions, case-based scenarios, embedded knowledge appraisal, and opportunities to meaningfully interact with presenters and peers.

**Conclusion:**

This study demonstrated positive and increasing prelicensure student ratings of knowledge acquisition and effective presentation methods across multifaceted learning sessions in an interfaculty pain curriculum. This study has implications for pain curriculum design aimed at promoting students' collaborative, patient-centered working skills.

**See commentary:** Trouvin A-P. “Ten-year mixed method evaluation of prelicensure health professional student self-reported learning in an interfaculty pain curriculum”: a view on pain education. PAIN Rep 2022;7:e1031.

Students attending learning sessions at the University of Toronto Interfaculty Pain Curriculum (2009–2019) in Toronto, Canada, self-report high ratings of knowledge acquisition and effective presentation methods.

## 1. Introduction

Pain is an increasingly prevalent problem worldwide with important physical, psychological, social, and economic implications.^39^ Unrelieved pain affects daily function, with negative consequences for physical and cognitive independence, relationships, employment, and quality of life.^50^ Pain has a significant impact on populations that routinely face greater health and social inequities such as women, indigenous people, gender diverse persons, people living in poverty, and older adults.^20^ Unrelieved pain is also one of the most common reasons for seeking medical assistance, thereby increasing the incremental costs of health care.^16^ As the provision of effective pain management may exceed the capacities of any one profession, interprofessional team-based pain care has emerged as a priority.^2^ Interprofessional approaches to pain management are believed to have the potential for improving implementation of new knowledge into practice and patient outcomes.

To support the availability of interprofessional team-based pain care, the International Association for the Study of Pain (IASP) has identified the importance of reshaping educational preparation of health care professionals.^17^ Recommended curricular considerations include a focus on collaborative, patient-centered working skills. Collaboration in this model is predicated on a reciprocal understanding of interprofessional team members' roles and responsibilities for assessing and treating pain.^45^ This approach requires students to be educated in interprofessional settings, so they have an opportunity to learn with, from, and about other health professionals' roles.^13^ Recommended content includes pain mechanisms, biopsychosocial concepts informing pain assessment and treatment, and the importance of patient or family engagement in care planning. The desired outcomes of these recommendations emphasize therapeutic skills in humanistic care and interprofessional collaboration.^27^

One example of interprofessional pain education based on IASP recommendations is the 20-hour interfaculty pain curriculum (IPC), developed by the University of Toronto Centre for the Study of Pain (UTCSP) in Toronto, Canada.^21^ The UTCSP-IPC is delivered over 3 consecutive days by faculty, research, and clinician members of the UTCSP and 4 partner health science faculties (Nursing, Dentistry, Pharmacy, and Medicine, which includes the Schools of Occupational Therapy, Physical Therapy, and the Physician Assistant Program).^48^ The UTCSP-IPC mirrors the tenets of the IASP curriculum recommendations in facilitating shared understandings of pain mechanisms, biopsychosocial treatment modalities, and interprofessional care planning. Two years after its inception in 2002, the IPC became a mandatory prelicensure curriculum in the participating faculties at the University of Toronto.

A curriculum delivery model suggested by the IASP is to balance large group lectures addressing core learning concepts (eg, pain mechanisms) with small group work (eg, collaborative pain care planning).^22^ Recent research has demonstrated the positive impact of this model on students' pain knowledge, beliefs, and interprofessional care plan quality through UTCSP- IPC participation.^7^ However, it is not yet clear how learners perceive knowledge acquisition in an interprofessional pain curriculum and in what ways delivery methods assist or hinder learning.^19^ An overreliance on cross-sectional reporting of summative learning measures omits opportunities to develop insights into how learning happens, from the perspective of students, and whether learning experiences are consistent.^10^ Given the significant human and economic investment in the design and execution of interprofessional pain education, additional research into the creation of collaborative working knowledge for health professional students is warranted.^32^

Social constructivist theory of learning and teaching proposes a solution to the above referenced knowledge gap. This theory asserts that students build knowledge through positive personal experiences and social interaction.^44^ The goal of curriculum evaluators using this approach would be to acquire multifaceted insights into student learning expectations, delivery preferences, engagement, and knowledge acquisition.^33^ Purposeful consideration of social constructivist factors when analyzing student feedback may yield design principles needed for the development of effective interprofessional pain education. Our aim, therefore, was to evaluate a decade of student perspectives on learning in an interfaculty pain curriculum to address gaps in our understanding of prelicensure pain education.

## 2. Methods

### 2.1. Design

We conducted a retrospective longitudinal mixed-method study to understand student perspectives in relation to pain knowledge acquisition and presentation effectiveness at the UTCSP-IPC over a 10-year period (2009–2019). Defined as research that involves collecting, analyzing, and integrating quantitative and qualitative data within a single study, mixed methods was chosen to enrich interpretive validity.^23^ The retrospective and longitudinal aspect was designed to uncover patterns of learning experience over time.^36^ In this evaluation, the quantitative data were given priority and the qualitative data were positioned to offer deeper insights into student learning experiences.

### 2.2. Curriculum overview

During the study period, the UTCSP-IPC was composed of 5 core learning sessions including self-study (online) and (small, medium, and large) in-person group learning formats: (1) 1 online self-study module on “pain mechanisms and manifestations,” (2) 1 online self-study module on “opioids as a component of pain management,” (3) 1 large group of patients and interprofessional panel, (4) 2 individually selected medium-group topical pain sessions, and (5) 1 small group facilitated interprofessional team-based pain care planning session (Table 1).^48^ In addition to the 5 core learning sessions, each faculty independently delivered and evaluated 1 uniprofessional learning session. Students were invited to provide quantitative and qualitative learning feedback for the 5 core learning sessions to the UTCSP each year.

**Table 1:** Overview of the 5 core learning components of the interprofessional pain curriculum.

Core learning session	Content and process	Student participation	Teaching–learning strategies
1. Self-study, online module	1-h long online module on foundational topics: *Pain Mechanisms and Manifestations*	Single students	Asynchronous online modules. The opioid module includes interprofessional and profession specific perspectives and is case-based
2. Self-study, online module	1-h long online module on foundational topics: *Opioids*	Single students	Asynchronous online modules. The opioid module includes interprofessional and profession specific perspectives and is case-based
3. Multiprofessional, large group session	3-h patient and interprofessional care team panel presentation demonstrating a person-centered approach to pain assessment and management, interprofessional collaboration, and communication in pain care	Approximately 500–1,000 students/y	Patient panel: people with lived experience (acute TMJ pain, acute and sickle-cell related pain, and chronic neuropathic pain) Interprofessional panel: interprofessional specialty pain team (physiatrist, nurse, physical therapist, and pharmacist) Panels are facilitated by IPC cochair and interaction facilitated with students using technology
4. Multiprofessional, medium group concurrent sessions	2 × 1.25-h presentations on “*Hot” Clinical Topics:* Addressing the current opioid crisis, issues, and challenges in cancer pain, headaches, pharmacology of pain, cannabis for pain, osteoarthritis, and mindfulness for pain management	Approximately 30–100 students/year per session depending on the student selection	Students select 2 didactic presentations of their choice from a menu of options Presenters from different professions for each topic, recognized as experts
5. Interprofessional, small group sessions	2 × 3-h interprofessional, team sessions to discuss virtual interactive case (VIC)-based examples of acute and persistent pain assessment and develop interprofessional pain management care plans	Interprofessional teams of 10 students each	Faculty-affiliated clinician and scientist facilitators

IPC, interfaculty pain curriculum; TMJ, temporomandibular joint; VIC, virtual interactive case.

### 2.3. Participants

Since its inception, approximately 1000 undergraduate health sciences students from the 4 participating health science faculties have attended the UTCSP-IPC annually. Yearly delivery was conditioned by space and resource availability and the willingness of faculties to situate the curriculum within their individual programs. Participants were full-time accelerated or second-entry undergraduate students with a previous degree from a variety of disciplines. Depending on the duration of their programs, students were in their second or third year of study at the time of participation. Ethical approval for all evaluative methods was obtained annually from the University of Toronto Research Ethics Board. Students were notified of the research component of the UTCSP-IPC evaluation, and consent to participate was voluntary. Survey identification numbers were automatically assigned by the online system to maintain student confidentiality.

### 2.4. Procedure and data collection methods

Students were automatically enrolled in the UTCSP-IPC through a computerized learning management system which provided a schedule of events, preparatory readings, and the opportunity to individually select topical pain learning sessions. At the completion of each day, students were provided with an email link to a Daily Content and Process Questionnaire (DCPQ), which invited them to rate their level of agreement across a range of statements including but not limited to the following: (1) knowledge acquisition and (2) effective presentation methods. Knowledge acquisition was defined as the degree to which new knowledge, understanding, or awareness was achieved. Effective presentation methods were defined as the degree to which the chosen learning format and/or session organization successfully supported learning. The response framework for each item consisted of a 5-point Likert scale: A (strongly disagree), B (disagree), C (neutral), D (agree), or E (strongly agree). Free-text comments were also invited to contextualize student feedback (Table 2).

**Table 2:** Daily content and process questionnaire example.

This session:	Strongly disagree	Disagree	Neutral	Agree	Strongly agree
1. Increased my knowledge of pain definitions, mechanisms, and classifications.	A	B	C	D	E
2. Increased my awareness of an integrated approach to the pain patient that incorporates biological, psychological, and social components.	A	B	C	D	E
3. Increased my knowledge of manifestations of acute and chronic pain.	A	B	C	D	E
4. Was effectively presented.	A	B	C	D	E
5. Was relevant to my profession.	A	B	C	D	E
6. Was free of commercial bias or influence.	A	B	C	D	E

Please circle the descriptor that most accurately describes your level of agreement with each statement: strongly disagree (A), disagree (B), neutral (C), agree (D), and strongly agree (E).

### 2.5. Quantitative analysis

Daily Content and Process Questionnaire items pertaining to knowledge acquisition and presentation effectiveness results were first identified for the 5 core learning sessions (2009–2019). Thereafter, descriptive statistics for DCPQ scores were computed for each core session cohort indicating the relative percentage of “agree or strongly agree” ratings, respectively. Pearson correlations (1-tailed) were performed to measure change over time (eg, years 2009–2019) for mean session rating scores. Data were analyzed using SPSS ver. 26 (IBM Corp. Released 2019. IBM SPSS Statistics for Windows, Version 26.0. Armonk, NY: IBM Corp). The statistical significance was set at *P* < 0.05.

### 2.6. Qualitative analysis

**Figure 1.**
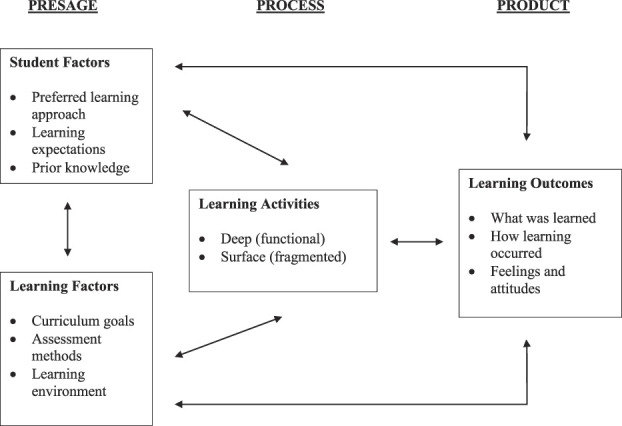
The 3-P social constructivist model of teaching and learning (modified).

Directed content analyses were conducted on students' narrative feedback collated for each core session cohort.^3^ For each session and year, paired investigators independently reviewed student narratives and coded them into affirmative (facilitator) and negative (barrier) categories for (1) knowledge acquisition and (2) effective presentation methods. Investigator pairs presented coding results to the full investigation team, and consensus was reached regarding categorization through discussion. The affirmative and negative results were subsequently mapped onto a modified version of the Bigg constructivist-alignment model of learning and teaching to provide a framework for understanding student expectations, experiences, and their impact on learning (Fig. 1).^14^ The Biggs modified model conceptualizes the learning process as an interactive system of 3 variables: presage factors (eg, learning expectations and learning context), process factors (eg, deep or surface learning experiences), and product factors (eg, learning achieved and affective outcomes). The model asserts that deep (eg, comprehensive) rather than surface (eg, fragmented) learning occurs where factors in each domain align for students.

### 2.7. Mixed-method analysis

In the final stage of the analysis, the quantitative and qualitative findings were integrated through joint displays, to draw out new insights beyond the information gleaned from the separate quantitative and qualitative results.^30^ Three possible outcomes were considered during this team-based analytic process: (1) *corroboration* when the findings from both types of data confirmed the other, (2) *expansion* when the findings from the 2 sources of data expanded insights into complementary or hitherto unknown aspects of the phenomenon, and (3) *discordance* when the findings of the qualitative and quantitative findings are inconsistent or contradicted one other.^11^

## 3. Results

A total of 10,693 students were enrolled in the UTCSP-IPC over the study period (2009–2019) with a mean annual attendance of 972 students (±SD:102). We obtained 10 years (2009–2019) of evaluable data for the patient and interprofessional panel, individually selected topical pain sessions, and facilitated interprofessional care team sessions. Six (2014–2019) and 5 (2015–2019) years of evaluable data were available for the “pain mechanisms and manifestations” and “opioids as a component of pain management” modules, respectively, due to their adaptation from large lecture sessions to online self-study formats and the incorporation of new evidence-based content.

### 3.1. Quantitative results

The mean annual number of DCPQ responses pertaining to 1) knowledge acquisition and (2) effective presentation methods was 335.8 and 317.2, respectively. This comprised ≥33% of the annual participating student cohorts. The mean proportion of students rating “agree or strongly agree” for knowledge acquisition and effective presentation methods across sessions were 79.3% (±SD:3.4) and 76.7% (±SD:6.0), respectively. Individual mean session ratings of “agree or strongly agree” for knowledge acquisition and effective presentation, respectively, included (1) online self-study pain mechanisms and manifestations module, 77.9% (SD:± 15.18) and 75.0% (±SD:16.1); (2) online self-study opioids module, 84.8% (SD:± 4.9) and 84.8% (SD:± 8.6); (3) patient and interprofessional panel, 76.8% (SD:± 6.2) and 69.8% (SD ± 13.2); (4) topical pain sessions, 81.1% (SD: ± 5.7) and 73.3% (SD: ± 13.3); and (5) facilitated interprofessional team-based pain care planning, 76.9% (SD:± 5.7) and 80.9% (SD ± 5.1). Knowledge acquisition scores increased with time for the online self-study pain mechanisms and manifestations module (*P* = 0.03; *r* = 0.735), online self-study opioid module (*P* = 0.04, *r* = 0.800), and individually selected topical pain sessions (*P* = 0.03; *r* = 0.266). Effective presentation scores increased with time for the online self-study pain mechanisms and manifestations module (*P* < 0.001; *r* = 0.976), online self-study opioid module (*P* = 0.019; *r* = 0.900), individually selected topical pain sessions (*P* < 0.001, *r* = 0.517), and patient or interprofessional panel sessions (*P* = 0.03, *r* = 0.391) (Table 3).

**Table 3:** Knowledge acquisition and presentation effectiveness scores (2009–2019).

Session	1. Pain mechanisms module	2. Opioids module	3. Patient and interprofessional panel	4. Topical pain sessions	5. Interprofessional team-based care planning
	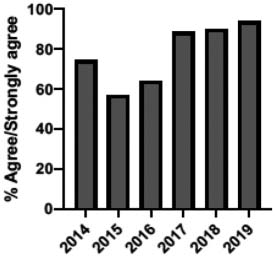	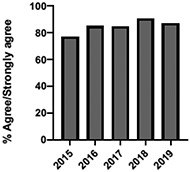	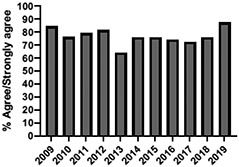	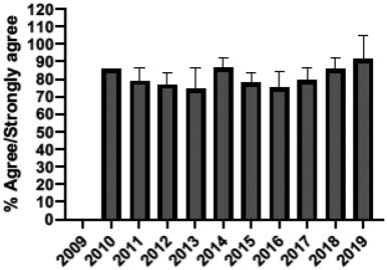	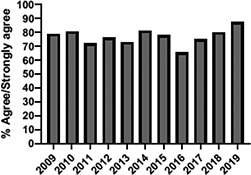
	1a. Knowledge acquisition	2a. Knowledge acquisition	3a. Knowledge acquisition	4a. Knowledge acquisition	5a. Knowledge acquisition
Mean score (%, SD)	**77.9 ± 15.18%** Min: 56.9% (2015) Max: 93.8% (2019)	**84.8 ± 4.9%** Min: 77.1% (2015) Max: 90.5% (2018)	**76.8 ± 6.2%** Min: 63.9% (2013) Max: 87.2% (2019)	**81.1 ± 5.7%** Min: 74.4% (2013) Max: 91.2% (2019)	**76.9 ± 5.7%** Min: 65.5% (2016) Max: 87.3% (2019)
Mean respondents (n, SD)	**328.5 ± 122.6** Min: 177 (2019) Max: 537 (2017)	**394.6 ± 185.6** Min: 177 (2019) Max: 588 (2017)	**246.9 ± 92.3** Min: 85 (2019) Max: 270 (2009)	**107.8 ± 59.5** Min: 39 (2017) Max: 216 (2010)	**601.5 ± 275.6** Min: 46 (2016) Max: 844 (2019)
Time Associations	***P* = 0.03; *r* = 0.735**	***P* = 0.04, *r* = 0.800**	***P* = 0.44; *r* = -0.030**	***P* = 0.03; *r* = 0.266**	***P* = 0.30; *r* = 0.172**
	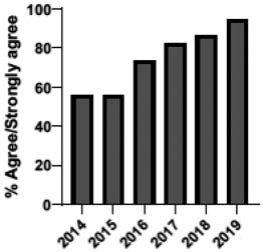	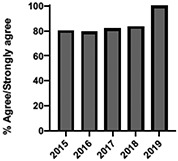	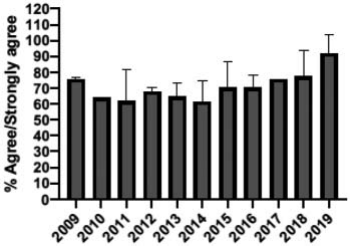	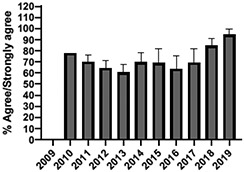	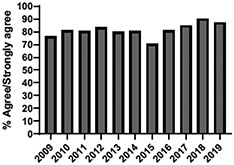
	1b. Presentation effectiveness	2b. Presentation effectiveness	3b. Presentation effectiveness	4b. Presentation effectiveness	5b. Presentation effectiveness
Mean score (%, SD)	**75.0 ± 16.1%** Min: 56.1% (2009) Max: 94.4% (2019)	**84.8 ± 8.6%** Min: 79.2% (2009) Max: 100.0% (2019)	**69.8 ± 13.2%** Min: 61.2% (2014) Max: 91.8% (2019)	**73.3 ± 13.3%** Min: 63.6% (2016) Max: 94.5% (2019)	**80.9 ± 5.1%** Min: 76.3% (2009) Max: 90.4% (2019)
Mean respondents (n, SD)	**328.3 ± 122.3** Min: 177 (2019) Max: 290 (2018)	**394.8 ± 83.1** Min: 177 (2019) Max: 589 (2015)	**245.5 ± 125.0** Min: 85 (2019) Max: 370 (2009)	**83.9 ± 64.4** Min: 44 (2017) Max: 216 (2010)	**533.5 ± 290.9** Min: 46 (2016) Max: 844 (2019)
Time Associations*	***P* = 0.00; *r* = 0.976**	***P* = 0.01; *r* = 0.900**	***P* = 0.03; *r* = 0.391**	***P* = 0.00; *r* = 0.517**	***P* = 0.11; *r* = 0.518**

* Statistical significance set at *P* < 0.05.

### 3.2. Qualitative results

A total of 2,400 student narratives were examined with a mean of 480 (±SD:186.7) statements attributed to each of the 5 core sessions for the study period (Table 4). Presage data identified that regardless of the session format or topic, students expected learning content to have relevance for clinical practice, clarify interprofessional roles in pain management, and transcend baseline pain knowledge:

“I will use what I learned today in future practice.”

“The interprofessional panel provided deeper insight into the connections between professions and their specific roles in the health care system.”

“[The] presentation was well-prepared, included new information, and improved my understanding of the interprofessional approach in pain management.”

**Table 4:** Qualitative results mapped to the 3-P framework.

Domain	1. Pain mechanisms and manifestations module	2. Opioids module	3. Patient and interprofessional panel	4. Topical pain sessions	5. Interprofessional team-based care planning
Size (n)	Single (1)	Single (1)	Large (500–1000)	Medium (30–100)	Small (10)
Presage					
Student factors • Preferred learning approach • Learning expectations • Prior knowledge	“I liked the engagement by clicking on different tabs to read more.” (facilitator)	“The case study was really informative and interactive.” (facilitator)	“Really enjoyed the morning session with the interaction between patients and interprofessional members discussing pain and its implications.” (facilitator)	“It was great to have the opportunity to choose which specific topics we were interested in learning about.” (facilitator)	"I really liked the interactivity of this session.” (facilitator)
	“The [module] interface or software was rather clumsy and slow.” (barrier)	“The module is a bit laggy, not sure if this was due to many people accessing it?” (barrier)	“The introduction of the patient panel sections did not offer any information I could apply to my clinical practice that I don't already know.” (barrier)	“Was not interactive at all. I would have appreciated if there was more participation encouraged.” (barrier)	"I would suggest making the session shorter.” (barrier)
Learning factors • Curriculum goals • Assessment methods • Learning environment	“This [module] has been very enlightening in terms of understanding the roles of other health professionals. (facilitator)	“Improved my understanding of the interprofessional approach in pain management.” (facilitator)	“The patient panel was interesting and thought provoking, and giving them a chance to share their story and answer questions was the highlight of the whole event.” (facilitator)	“All presenters were very engaging, and I really saw the interprofessional aspects of their talks.” (facilitator)	"Very efficient use of patient cases and stories [which] are quite powerful.” (facilitator)
	“A little more time should have been put into editing the question set.” (barrier)	“I would have liked to learn more about the dangers surrounding opioid use.” (barrier)	"Venue was not really conducive to my learning—I couldn't hear or see for certain portions of the presentation.” (barrier)	"It was too general. Practical take-aways that could be applied to one's profession would be more useful.” (barrier)	“Our group missed the point of interacting to learn interprofessionally and focused too much on completing the assignment.” (barrier)
Process					
Deep learning	“Overall this module was excellent—well put together visually, well-organized, and with a flow through that made sense.” (facilitator)	“The interactive module solidified key points covered in lectures. The cases were practical and stimulated rational thinking in approaching pain.” (facilitator)	“I really enjoyed listening to the interprofessional team panel because they really brought together how interprofessional care works. Until today, most of the other sessions showed the patient managing the system alone. This team showed how in one setting you can have a variety of professionals working collaboratively.” (facilitator)	“Very interesting session that helped me to better understand the overall debate on cannabis use and when it may be appropriate.” (facilitator)	"This was a very helpful session. I learned a lot about the other health care professionals, their specific roles, and how to best communicate in a large interdisciplinary team.” (facilitator)
Surface learning	“The amount of material presented was a little overwhelming so at times I was skimming the material instead of reading in-depth.” (barrier)	“I think I left understanding more terms, but not knowing how they fit together.” (barrier)	“The panel was a good idea. Did not learn about appropriate pain management and the interprofessional team and how we can work together.” (barrier)	“Practical take-aways that could be applied to one's profession would be more useful.” (barrier)	“The entire focus of the group ended up being “just getting things done.” (barrier)
Learning outcomes					
Knowledge • What was learned	“Module increased my knowledge of how important it is to treat pain before it becomes chronic.” (facilitator)	“I did not previously know that minor dental surgery generally requires anti-inflammatories and rarely opioids.” (facilitator)	“Having a panel of experts from a pain clinic showed how well these pain experts can work together for patient well-being” (facilitator)	“Did not know much at all before the session and now I know something about the topic.” (facilitator)	“Helped me gain insight with what other health care professionals do.” (facilitator)
	“For those with a background in pain, this is repetitive and not necessarily a good use of time.” (barrier)	“I didn't really find the module useful. It didn't really provide any information that I didn't already know.” (barrier)	"There wasn't anything here that I hadn't already learned in the (faculty) program" (barrier)	“Would like to hear the clear roles of nursing or medicine or pharm, etc.” (barrier)	“Rather than build my knowledge, it was more explaining and sharing my knowledge to all of the other professions.” (barrier)
Process • How learning occurred	“The diagrams and videos were also very helpful in synthesizing material in a visual way that kept up attention and increased deeper learning.” (facilitator)	“The answers to the quiz questions were really thorough but also easy to understand and reinforced what the case studies were trying to portray. (facilitator)	“Very efficient use of patient cases and patient stories are quite powerful” (facilitator)	“[Presenter] amazing at explaining the learning objective and answering the audience questions.” (facilitator)	“The best education was in the informal discussions with my small group.” (facilitator)
	“At times, it felt like too much information was provided—possibly cut down to emphasize the main learning point.” (barrier)	“Questions throughout module tested information not yet introduced or never introduced.” (barrier)	“Not very engaging. Would have preferred more conversational style and more about the case.” (barrier)	“[Topic] could be even more effectively presented if the time was used to provide more clinical pearls.” (barrier)	“Some of the facilitated group discussion felt forced. I would have enjoyed a free form discussion.” (barrier)
Affective • Feelings and attitudes	“Amazing chronic pain coverage. This is eye-opening and not covered in depth in our program.” (facilitator)	“I believe that I have developed an increased respect for the level of knowledge and responsibilities of my peers in other faculties.” (facilitator)	"Developing a sense of empathy forms a crucial part of our dealing with patients presenting with any type of pain” (facilitator)	“[The] session has provided me with new dimension to see the pain and ways to handle such human suffering.” (facilitator)	“Made me truly reflect on the importance of IP practice and value each profession's unique roles” (facilitator)
	“Information was extremely dense, and it is a misrepresentation to report [it] can be completed in 1 h” (barrier)	“There was not enough detail. This module barely brushed the surface of this complex issue.” (barrier)	“It was very hard to absorb any information or just focus in general […] I think there should have been a balance between lecture style or interactive work today.” (barrier)	“I found the topics to be very biased toward medical and pharmaceutical management.” (barrier)	“I feel as though my time and profession was very disrespected by many of the students who had not had clinical experience” (barrier)

Contextual factors powerfully influencing student learning included the session size and comfort in the provided learning space (sightlines, sound, temperature, and nutrition breaks):

“The [large group] session was interesting; however, due to the size and layout of the room, it was difficult to hear. At the back of the room, there were students being very disrespectful and continually talking.”

For many students, distractions in larger group sessions negatively affected the quality of content engagement, ability to pose questions, and perception of time. By contrast, students positively reported on opportunities to individually select medium-size group learning sessions and overall greater ease posing questions in smaller groups:

“Overall I learned a lot of new knowledge but felt that the [large group] presentation was too long and did not allow for questions.”

“The most valuable parts were the [medium-size group] activities where you could choose the topics you were interested in, as well as the [small group] case-based activity.”

“Excellent practical information and case study examples [in individually selected sessions]. Great dialogue in question or answer periods.”

In addition to adequate discussion or question periods, process activities that best facilitated deep learning included the use of case-based scenarios, embedded knowledge appraisal feedback (eg, quizzes), and diversity of patient or professional representation among presenters:

“The cases were interesting and relevant to real practice. The cases were able to incorporate multidisciplinary approaches in helping the patient heal and relieve pain. Overall the module was enjoyable and informative.”

“I also liked how when you chose the answer, there was an explanation of why that answer was correct [or not].”

“Loved having the Nurse Practitioner and Social Worker present. A representative from Occupational Therapy or Physiotherapy would be great too.”

Product data indicated that students acquired new knowledge from one another, patient or panel members, and through opportunities to consolidate old and new knowledge. Some students described how patient presence in the curriculum recentered the learning experience and accelerated understand of partnership with patients in pain management:

“The patients were the real focus, and I appreciated the opportunity to hear from them on multiple occasions. It really helped in understanding the specific but pervasive issues of managing both acute and chronic pain […] and the importance of validating the struggle.”

Affective outcomes included empathic feelings towards patients, positive self-appraisal of performance in a team setting, and value accorded to interprofessional practice:

“Not only was the session informative, it was also humbling and made me very empathetic towards what these people have gone through.”

“Helped to build my confidence, reinforced that I have ideas to contribute to the team.”

“Helped me most in terms of understanding the importance of approaching pain from an IP [interprofessional] perspective.

Whereas most students reported positive learning experiences and outcomes, a few students reported negative feelings associated with perceived disagreement between patient or professional panel members, insufficient collaboration among student teams, and facilitator feedback methods:

“Students are well aware of [patient–provider] conflict. It would be more fruitful for the facilitator to not be so leading [and] address that conflict occurs and instead of using a case study, use real-world experiences to describe how conflict would play out, and how it affects a patient's experience.”

“As always, a few [student team members] do nothing and others carry the weight. Smaller groups would be much easier.”

To circumvent conflict in interactive sessions, students recommended advanced facilitator preparation and smaller team sizes to enhance collaborative work.

### 3.3. Mixed-method results

The qualitative data corroborated the quantitative findings of knowledge acquisition and expanded insights into student expectations for the utility of learning for practice and clarification of interprofessional roles across the 5 core sessions. Discordance was identified in circumstances where presentation content was perceived as review material, lacking in supporting evidence, or did not clarify interprofessional roles in pain management, thereby limiting knowledge development for practice. The qualitative data corroborated the quantitative findings of effective presentation methods. Expansion was identified by student preferences for esthetically pleasing online self-study learning modules and small-to-moderate sized in-person sessions (<100 students), which accommodated meaningful interaction with presenters and peers. Discordance regarding presentation effectiveness was identified in circumstances where students perceived sessions to be unnecessarily long, opportunities for social interaction were limited, and facilitators underprepared.

## 4. Discussion

In this study, aiming to address gaps in our understanding of learning about pain in an interfaculty curriculum, we identified high mean prelicensure student agreement for knowledge acquisition or effective presentation ratings across 5 core learning sessions. Session ratings increased over time for online self-study, individually selected topical, and patient or interprofessional panel sessions. Using a constructivist framework applied to qualitative data, we found prelicensure health professional student perceptions of new and clinically relevant information, clarity regarding interprofessional roles and responsibilities in pain management, and a diverse mix of patient and professional representation among presenters to be associated with knowledge acquisition. Presentation effectiveness was associated with the smaller session size, individually selected sessions, use of case-based scenarios, embedded knowledge appraisal, and opportunities to meaningfully interact with presenters and peers.

Our findings are congruent with the current literature, namely, that interprofessional education is well received by health professional students^38^ and that, on average, a multifaceted pain curriculum intervention offers durable results with respect to student knowledge acquisition.^7^ The qualitative findings contribute additional insights regarding the value and affective impact of the curriculum such as the development of empathy for patients and improved confidence in collaborative team-based performance. Our findings align with prior research demonstrating that empathy can be actualized or enhanced through educational experiences.^25^ This result may be important for counteracting an observed trend in medical education where students report reduced communication, concern, and empathy for chronic pain patients as they progress through their training.^40^

Consistent knowledge acquisition ratings in this study may be explained through social constructivist theory of learning and teaching which situates students as active rather than passive participants in the learning process.^7^ The curriculum is updated annually with a focus on active learning strategies, which may explain rating improvement over time.^29^ Growing societal awareness of chronic pain as a public health emergency may also have contributed to rating improvement.^20^ Study results support IASP recommendations for small group work^22^ and align with research demonstrating improved social interaction, learning scores, and student satisfaction in smaller groups.^1,26^ It is plausible that psychological safety conferred by small groups (ie, students feel comfortable asking questions) may contribute to knowledge exchange.^46^ Regardless of the session size and delivery mode, however, students in this study indicated that opportunities for interaction enhanced their engagement in learning.

We found students to have consistent expectations for the presentation of new, clinically relevant, and evidence-based information to support their professional development. Research on perspectives on learning among health professions students has emphasized the importance placed on the consolidation of skills,^47^ development of clinical reasoning, theory or practice integration,^34^ and nurturing of professional identity.^31^ Student perspectives of other interprofessional pain education activities also point to value placed on the consolidation of learning and promotion of clinical reasoning.^5^ This aligns with the constructivist idea that students strive to build on prior knowledge to develop a sense of competency. Findings suggest students may not perceive learning with reinforcement of prior knowledge (ie, review) alone, although student's ability to self-assess may not be accurate.^15^ Providing opportunities for the selection of topics may improve engagement in learning and optimize students' motivation to acquire new knowledge.

Students reported how case-based learning scenarios and embedded knowledge evaluation significantly enhanced their learning experience. Students commented on the degree of interest afforded through authentic case-study vignettes which are theorized to advance deep learning by emphasizing the direct relevance or application of new information for real-world clinical practice.^43^ Positive student responses to embedded knowledge evaluation in the form of quizzes may also have contributed to high knowledge acquisition ratings. As a form of self-appraisal of knowledge development, embedded quizzes can provide students with step-wise, personalized feedback throughout a course and have been reported to enhance learning retention, satisfaction, and final examination performance.^24^ Embedded quizzes applied to patient case-study vignettes may provide students with active opportunities to apply knowledge and practice problem solving.

Given student feedback regarding the importance of diversity of patient or professional participation in the curriculum, reflection on both representation and issues of equity should be considered in building pain learning curricula.^4^ Students recognize and acknowledge that social inequities are deeply entrenched in health care contexts where individuals representing marginalized communities are often disproportionately affected, as noted in statistics related to burden of disease and associated poorer outcomes.^28^ These considerations intersect with disparities in access to pain treatment across marginalized populations including women, indigenous people, gender diverse persons, people living in poverty, older adults, and those with mental health or substance use challenges.^8^ Thus, the pain curriculum is an important opportunity to demonstrate how marginalization amplifies the negative impacts of pain and how collaborative pain treatment can be an equity-oriented response to a public health problem.^49^

Our results showed students' improved understanding of the roles of other health professionals and willingness to work in a team-based approach to managing pain. When students from different professional programs work collaboratively to respond to the challenge of pain, they may be intrinsically motivated to work harder when group success depends on their collective contributions.^6^ We might theorize that perceptions of collaborative competency may be best developed within groups perceived to include a real-world mix of health professionals. In this context, students begin to understand the roles and scopes of practice as well as professional culture of different health care providers. Small working groups may alert students to the necessary resources for future collaboration in pain management including work coordination and the management of conflict.^41^ This raises the importance of facilitator roles in guiding students, managing their behaviours (eg, dominant or quiet behaviours), and acting as a role model.

Implementing knowledge translation innovations, such as this large educational initiative, is highly context dependent.^18^ Beyond the innovation, the process of implementation, the implementor, and contextual determinants (barriers and facilitators) need to be considered.^9^ For example, potential barriers to effective implementation of the UTCSP-ICP include financial and personnel resources. Costs and faculty resources required to offer this educational strategy (up until the past 2–3 years when the curriculum went online) have represented an annual financial and personnel challenge. Implementation success has been realized given potential modifiable and nonmodifiable barriers have been identified and overcome, largely because of the support from faculties and the university and the generosity of individuals volunteering their time and expertise. However, self-reported ratings of knowledge acquisition and the effectiveness of presentation methods constitute a limited view of implementation effectiveness. Additional investigation of implementation outcomes including feasibility, fidelity, implementation cost, reach, and sustainability will help to identify what works best across situations and settings and how best to promote spread and dissemination.^37^

Strengths of our study include mixed-method data integration, longitudinal evaluation, use of theory, and an interprofessional team of analysts.^12^ The mean number of annual student responses to knowledge acquisition and effective presentation questions falls within the reported response range among health professional trainees^35^ and clinicians.^42^ However, we could not establish characteristics of respondents and reasons for nonparticipation. Data drawn from students attending one university-based curriculum limits the extent to which these findings can be generalized to other students and settings. In addition, although some students readily benefited from the multifaceted and interactive curriculum, additional time or session modalities may be needed to improve their learning.

## 5. Conclusion

In a 10-year retrospective longitudinal study of the UTCSP-IPC, we found high mean ratings of prelicensure student agreement regarding knowledge acquisition to be correlated with perceived receipt of new and clinically relevant information and improved understanding of interprofessional roles and responsibilities in pain management. High mean ratings of presentation effectiveness corresponded with smaller session size, individually selected sessions, case-based scenarios, embedded knowledge appraisal, and opportunities to meaningfully interact with presenters and peers. Results offer theoretically informed design principals needed for the development of effective interprofessional pain education and evidence that a multifaceted curriculum intervention provides durable results.

## Disclosures

C. M. Dale was supported by University of Toronto Centre for the Study of Pain (UTCSP Scientist), the Canadian Institutes of Health Research (CIHR), and Sunnybrook Health Sciences Centre. I. Cioffi was supported by the University of Toronto Centre for the Study of Pain (UTCSP Scientist) and the Faculty of Dentistry at the University of Toronto.The remaining authors have no conflicts of interest to declare.

## References

[1] Abu-Rish E Kim S Choe L Varpio L Malik E White AA Craddick K Blondon K Robins L Nagasawa P Thigpen A Chen L-L Rich J Zierler B. Current trends in interprofessional education of health sciences students: a literature review. J Interprof Care 2012;26:444–51. 22924872 10.3109/13561820.2012.715604 PMC7594101

[2] Arwood E Rowe JM Singh NS Carr DB Herr KA Chou R. Implementing a paradigm shift: incorporating pain management competencies into pre-licensure curricula. Pain Med 2015;16:291–300. 25244226 10.1111/pme.12563

[3] Bengtsson M. How to plan and perform a qualitative study using content analysis. NursingPlus Open 2016;2:8–14.

[4] Bhutta ZA Chen L Cohen J Crisp N Evans T Fineberg H Frenk J Garcia P Horton R Ke Y Kelley P Kistnasamy B Meleis A Naylor D Pablos-Mendez A Reddy S Scrimshaw S Sepulveda J Serwadda D Zurayk H. Education of health professionals for the 21st century: a global independent Commission. Lancet 2010;375:1137–8. 20362799 10.1016/S0140-6736(10)60450-3

[5] Burgess A Kalman E Haq I Leaver A Roberts C Bleasel J. Interprofessional team-based learning (TBL): how do students engage? BMC Med Educ 2020;20:118. 32306968 10.1186/s12909-020-02024-5 PMC7168950

[6] Carney PA Thayer EK Palmer R Galper AB Zierler B Eiff MP. The benefits of interprofessional learning and teamwork in primary care ambulatory training settings. J Interprofessional Educ Pract 2019;15:119–26.

[7] Cioffi I Dale CM Murphy L Langlois S Musa R Stevens B. Ten years of interfaculty pain curriculum at the University of Toronto: impact on student learning. Pain Rep 2021;6:e974. 34870057 10.1097/PR9.0000000000000974 PMC8635288

[8] Craig KD Holmes C Hudspith M Moor G Moosa-Mitha M Varcoe C Wallace B. Pain in persons who are marginalized by social conditions. PAIN 2020;161:261–5. 31651578 10.1097/j.pain.0000000000001719 PMC6970566

[9] Damschroder LJ Aron DC Keith RE Kirsh SR Alexander JA Lowery JC. Fostering implementation of health services research findings into practice: a consolidated framework for advancing implementation science. Implement Sci IS 2009;4:50. 19664226 10.1186/1748-5908-4-50 PMC2736161

[10] Darlow B Brown M Gallagher P Gray L McKinlay E Purdie G Wilson C Pullon S Study Group LIP. Longitudinal impact of interprofessional education on attitudes, skills and career trajectories: a protocol for a quasi-experimental study in New Zealand. BMJ Open 2018;8:e018510. 10.1136/bmjopen-2017-018510 PMC5781053 29358432

[11] Fetters MD Curry LA Creswell JW. Achieving integration in mixed methods designs-principles and practices. Health Serv Res 2013;48:2134–56. 24279835 10.1111/1475-6773.12117 PMC4097839

[12] Fielding NG. Triangulation and mixed methods designs: data integration with new research technologies. J Mix Methods Res 2012;6:124–36.

[13] Fishman SM Young HM Lucas Arwood E Chou R Herr K Murinson BB Watt-Watson J Carr DB Gordon DB Stevens BJ Bakerjian D Ballantyne JC Courtenay M Djukic M Koebner IJ Mongoven JM Paice JA Prasad R Singh N Sluka KA St Marie B Strassels SA. Core competencies for pain management: results of an interprofessional consensus summit. Pain Med Malden Mass 2013;14:971–81. 10.1111/pme.12107 PMC3752937 23577878

[14] Freeth D Reeves S. Learning to work together: using the presage, process, product (3P) model to highlight decisions and possibilities. J Interprof Care 2004;18:43–56. 14668101 10.1080/13561820310001608221

[15] Gabbard T Romanelli F. The accuracy of health professions students' self-assessments compared to objective measures of competence. Am J Pharm Educ 2021;85:8405. 34283796 10.5688/ajpe8405 PMC8086612

[16] Goldberg DS McGee SJ. Pain as a global public health priority. BMC Public Health 2011;11:770. 21978149 10.1186/1471-2458-11-770 PMC3201926

[17] Gordon DB Watt-Watson J Hogans BB. Interprofessional pain education-with, from, and about competent, collaborative practice teams to transform pain care. Pain Rep 2018;3:e663. 29922750 10.1097/PR9.0000000000000663 PMC5999416

[18] Grimshaw JM Eccles MP Lavis JN Hill SJ Squires JE. Knowledge translation of research findings. Implement Sci IS 2012;7:50. 22651257 10.1186/1748-5908-7-50 PMC3462671

[19] Guraya SY Barr H. The effectiveness of interprofessional education in healthcare: a systematic review and meta-analysis. Kaohsiung J Med Sci 2018;34:160–5. 29475463 10.1016/j.kjms.2017.12.009 PMC12977169

[20] Health Canada. Canadian pain task force report: march 2021. 2021. Available at: https://www.canada.ca/en/health-canada/corporate/about-health-canada/public-engagement/external-advisory-bodies/canadian-pain-task-force/report-2021.html. Accessed February 2, 2022.

[21] Hunter J Watt-Watson J McGillion M Raman-Wilms L Cockburn L Lax L Stinson J Cameron A Dao T Pennefather P Schreiber M Librach L Kavanagh T Gordon A Cullen N Mock D Salter M. An interfaculty pain curriculum: lessons learned from six years experience. PAIN 2008;140:74–86. 18774226 10.1016/j.pain.2008.07.010

[22] IASP interprofessional pain curriculum outline. Int Assoc Study Pain IASP. Available at: https://www.iasp-pain.org/education/curricula/iasp-interprofessional-pain-curriculum-outline/. Accessed February 2, 2022.

[23] Johnson RB Onwuegbuzie AJ. Mixed methods research: a research paradigm whose time has come. Educ Res 2004;33:14–26.

[24] Khanna MM. Ungraded pop quizzes: test-enhanced learning without all the anxiety. Teach Psychol 2015;42:174–8.

[25] Kiosses VN Karathanos VT Tatsioni A. Empathy promoting interventions for health professionals: a systematic review of RCTs. J Compassionate Health Care 2016;3:7.

[26] Lairamore C Morris D Schichtl R George-Paschal L Martens H Maragakis A Garnica M Jones B Grantham M Bruenger A. Impact of team composition on student perceptions of interprofessional teamwork: a 6-year cohort study. J Interprof Care 2018;32:143–50. 29131704 10.1080/13561820.2017.1366895

[27] van Lankveld W Afram B Staal JB van der Sande R. The IASP pain curriculum for undergraduate allied health professionals: educators defining competence level using Dublin descriptors. BMC Med Educ 2020;20:60. 32111209 10.1186/s12909-020-1978-z PMC7048028

[28] Lavizzo-Mourey RJ Besser RE Williams DR. Understanding and mitigating health inequities—past, current, and future directions. N Engl J Med 2021;384:1681–4. 33951376 10.1056/NEJMp2008628

[29] Michael J. Where's the evidence that active learning works? Adv Physiol Educ 2006;30:159–67. 17108243 10.1152/advan.00053.2006

[30] O'Cathain A Murphy E Nicholl J. Three techniques for integrating data in mixed methods studies. BMJ 2010;341:c4587. 20851841 10.1136/bmj.c4587

[31] O'Doherty D Culhane A O'Doherty J Harney S Glynn L. McKeague* H, Kelly* D. Medical students and clinical placements - a qualitative study of the continuum of professional identity formation. Educ Prim Care 2021;32:202–10. 33583348 10.1080/14739879.2021.1879684

[32] Olson R Bialocerkowski A. Interprofessional education in allied health: a systematic review. Med Educ 2014;48:236–46. 24528458 10.1111/medu.12290

[33] Palincsar AS. Social constructivist perspectives on teaching and learning. Annu Rev Psychol 1998;49:345–75. 15012472 10.1146/annurev.psych.49.1.345

[34] Paterson J Higgs J Wilcox S Villeneuve M. Clinical reasoning and self-directed learning: key dimensions in professional education and professional socialisation. Focus Health Prof Educ 2002;4:5–21.

[35] Phillips AW Friedman BT Utrankar A Ta AQ Reddy ST Durning SJ. Surveys of health professions trainees: prevalence, response rates, and predictive factors to guide researchers. Acad Med J Assoc Am Med Coll 2017;92:222–8. 10.1097/ACM.0000000000001334 27532869

[36] Plano Clark VL Anderson N Wertz JA Zhou Y Schumacher K Miaskowski C. Conceptualizing longitudinal mixed methods designs: a methodological review of health sciences research. J Mix Methods Res 2015;9:297–319.

[37] Proctor E Silmere H Raghavan R Hovmand P Aarons G Bunger A Griffey R Hensley M. Outcomes for implementation research: conceptual distinctions, measurement challenges, and research agenda. Adm Pol Ment Health 2011;38:65–76. 10.1007/s10488-010-0319-7 PMC3068522 20957426

[38] Reeves S Zwarenstein M Goldman J Barr H Freeth D Koppel I Hammick M. The effectiveness of interprofessional education: key findings from a new systematic review. J Interprof Care 2010;24:230–41. 20178425 10.3109/13561820903163405

[39] Rice ASC Smith BH Blyth FM. Pain and the global burden of disease. Pain 2016;157:791–6. 26670465 10.1097/j.pain.0000000000000454

[40] Rice K Ryu JE Whitehead C Katz J Webster F. Medical trainees' experiences of treating people with chronic pain: a lost opportunity for medical education. Acad Med 2018;93:775–80. 29140917 10.1097/ACM.0000000000002053 PMC5929494

[41] Schot E Tummers L Noordegraaf M. Working on working together. A systematic review on how healthcare professionals contribute to interprofessional collaboration. J Interprof Care 2020;34:332–42. 31329469 10.1080/13561820.2019.1636007

[42] Taylor T Scott A. Do physicians prefer to complete online or mail surveys? Findings from a national longitudinal survey. Eval Health Prof 2019;42:41–70. 30384770 10.1177/0163278718807744

[43] Thistlethwaite JE Davies D Ekeocha S Kidd JM MacDougall C Matthews P Purkis J Clay D. The effectiveness of case-based learning in health professional education. A BEME systematic review: BEME Guide No. 23. Med Teach 2012;34:e421–44. 22578051 10.3109/0142159X.2012.680939

[44] Thomas A Menon A Boruff J Rodriguez AM Ahmed S. Applications of social constructivist learning theories in knowledge translation for healthcare professionals: a scoping review. Implement Sci IS 2014;9:54. 24885925 10.1186/1748-5908-9-54 PMC4040365

[45] Thompson K Johnson MI Milligan J Briggs M. Twenty-five years of pain education research-what have we learned? Findings from a comprehensive scoping review of research into pre-registration pain education for health professionals. PAIN 2018;159:2146–58. 30052574 10.1097/j.pain.0000000000001352

[46] Tsuei SH-T Lee D Ho C Regehr G Nimmon L. Exploring the construct of psychological safety in medical education. Acad Med J Assoc Am Med Coll 2019;94:S28–35. 10.1097/ACM.0000000000002897 31365407

[47] Tucker K Wakefield A Boggis C Lawson M Roberts T Gooch J. Learning together: clinical skills teaching for medical and nursing students. Med Educ 2003;37:630–7. 12834421 10.1046/j.1365-2923.2003.01558.x

[48] University of Toronto Centre for the study of pain. Available at: http://sites.utoronto.ca/pain/research/interfaculty-curriculum.html. Accessed February 2, 2022.

[49] Wallace B Varcoe C Holmes C Moosa-Mitha M Moor G Hudspith M Craig KD. Towards health equity for people experiencing chronic pain and social marginalization. Int J Equity Health 2021;20:53. 33531018 10.1186/s12939-021-01394-6 PMC7852178

[50] Wilson MG Lavis JN Ellen ME. Supporting chronic pain management across provincial and territorial health systems in Canada: findings from two stakeholder dialogues. Pain Res Manag 2015;20:269–79. 26291124 10.1155/2015/918976 PMC4596635

